# Chasing Sequencing Perfection: Marching Toward Higher Accuracy and Lower Costs

**DOI:** 10.1093/gpbjnl/qzae024

**Published:** 2024-03-11

**Authors:** Hangxing Jia, Shengjun Tan, Yong E Zhang

**Affiliations:** CAS Key Laboratory of Zoological Systematics and Evolution & State Key Laboratory of Integrated Management of Pest Insects and Rodents, Institute of Zoology, Chinese Academy of Sciences, Beijing 100101, China; CAS Key Laboratory of Zoological Systematics and Evolution & State Key Laboratory of Integrated Management of Pest Insects and Rodents, Institute of Zoology, Chinese Academy of Sciences, Beijing 100101, China; CAS Key Laboratory of Zoological Systematics and Evolution & State Key Laboratory of Integrated Management of Pest Insects and Rodents, Institute of Zoology, Chinese Academy of Sciences, Beijing 100101, China; University of Chinese Academy of Sciences, Beijing 100049, China; CAS Center for Excellence in Animal Evolution and Genetics, Chinese Academy of Sciences, Kunming 650223, China

**Keywords:** Sequencing error, High-fidelity sequencing, Consensus sequencing, Single-molecule sequencing, Rare mutation

## Abstract

Next-generation sequencing (NGS), represented by Illumina platforms, has been an essential cornerstone of basic and applied research. However, the sequencing error rate of 1 per 1000 bp (10^−3^) represents a serious hurdle for research areas focusing on rare mutations, such as somatic mosaicism or microbe heterogeneity. By examining the high-fidelity sequencing methods developed in the past decade, we summarized three major factors underlying errors and the corresponding 12 strategies mitigating these errors. We then proposed a novel framework to classify 11 preexisting representative methods according to the corresponding combinatory strategies and identified three trends that emerged during methodological developments. We further extended this analysis to eight long-read sequencing methods, emphasizing error reduction strategies. Finally, we suggest two promising future directions that could achieve comparable or even higher accuracy with lower costs in both NGS and long-read sequencing.

## Introduction

Massive parallel short-read sequencing, or next-generation sequencing (NGS), technologies have revolutionized basic and applied biological research. However, sequencing errors have limited their broader application. Among various sequencing platforms, Illumina provides a high sequencing accuracy quantified by error rate, which reaches 1 per 1000 bp or 10^−3^ [1,2]. Such error rate is often represented by the Phred quality score or Q score [3,4], which is defined as −10 × log_10_ error rate, and thus 10^−3^ is equivalent to Q30. Q30 is adequate for many applications, but not sufficient for detecting and quantifying rare mutations (frequency < 0.1%) in heterogeneous mixtures of cells or DNA molecules. When conducting investigations in somatic or germline cell populations, microbe populations, and forensic samples, the allele frequency of target mutations could be much lower than 10^−3^ [5–8]. For example, the human somatic mutation rate of different tissues is between 10^−9^ to 10^−8^ per bp per year [9,10], which is higher than the germline mutation rate (10^−10^ per bp per year or 10^−8^ per bp per generation) [11]. Given the low signal-to-noise ratio, the *bona fide* mutations would be buried in the sea of sequencing errors. Thus, if the detection limit or sensitivity is pushed to 10^−9^, the error rate should be even lower [5,6]. Motivated by the demand for detecting rare mutations in fields such as somatic or germline mosaicism, cancer genomics, antibiotic resistance, or forensics [5,8,12], numerous high-fidelity NGS methods targeting single DNA molecules have been developed since 2011. Although these methods are generally associated with higher sequencing costs, they reach an error rate ranging from 10^−9^ to 10^−6^ [5,6,9,13–19].

Two comprehensive reviews, published in 2018, extensively covered high-fidelity NGS methods and application directions [5,6]. Our intention is not merely to provide an update on the expanding methodological reservoir. Instead, we aim to summarize the major causes of sequencing errors, and present a novel framework by extracting key error-mitigating strategies and redefining existing methods through these strategies. We further describe high-fidelity long-read sequencing. Through such a review of methodological development spanning the past 12 years, we ultimately propose promising future directions for achieving even more precise short-read or long-read sequencing at reduced costs.

## Three major causes of sequencing errors

**Figure 1:**
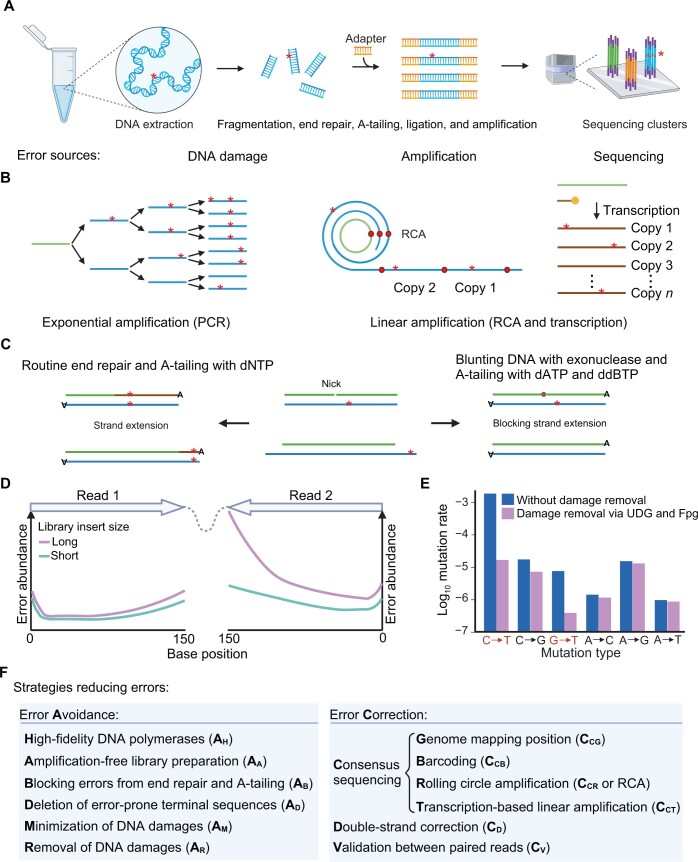
Causes underlying sequencing errors and strategies reducing errors **A**. An overall schema of the typical Illumina sequencing process. The left, middle, and right images show DNA extraction, library construction, and sequencing, respectively. Notably, in the right image, three Illumina sequencing clusters are shown, each of which consists of PCR products from one single DNA molecule. Through sequencing by synthesis of the PCR products within each cluster, Illumina reports a cluster-level consensus as the final data. Errors (indicated by red stars) can happen in each step and can be roughly divided into three types: DNA damage, PCR-associated errors, and sequencing-associated errors. **B**. Two types of amplification modes. Errors are again indicated by red stars. The DNA template is marked in green, while amplified DNA products are shown in blue. In the middle image, the red dots mark the boundary of each copy. In the right image, RNA is marked in brown, while RNA polymerase is shown as an orange dot. **C**. Error avoidance during end repair or A-tailing. The two DNA strands are represented in green and blue, while the extended single-strand DNA is shown in brown. Errors are indicated by red stars. The central image illustrates internal (top) and terminal (bottom) errors. The left image showcases standard end repair and A-tailing, and the right image displays DNA blunting and modified A-tailing. In the left image, internal (top) and terminal (bottom) errors propagate to the complementary strand during end repair and A-tailing. In the top panel of the right image, the internal nick is extended according to the complementary strand; if ddBTP (ddGTP, ddCTP, or ddTTP; shown as a brown dot) is added, the extension stops. In the bottom panel of the right image, exonuclease is used to cut the single-strand overhang, followed by the addition of dATP. **D**. The sequencing error distribution of Illumina paired-end sequencing (2 × 150 bp) in libraries with short and long insert sizes. Position 0 marks the 5′ terminal of reads. This figure is modified from [27]. **E**. Mutation signature with or without removal of DNA damage. DNA damages induce C-to-T and G-to-T errors (marked in red), which can be removed by UDG and Fpg, respectively. This figure is modified from [23]. **F**. Strategies for reducing sequencing errors. Two types of strategies could be further divided into 12 subtypes. PCR, polymerase chain reaction; ddGTP, 2′,3′-dideoxyguanosine 5′-triphosphate; ddCTP, 2′,3′-dideoxycytidine 5′-triphosphate; ddTTP, 2′,3′-dideoxythymidine 5′-triphosphate; dATP, deoxyadenosine triphosphate; UDG, uracil-DNA glycosylase; Fpg, formamidopyrimidine DNA glycosylase; C, cytosine; T, thymine; G, guanine; A, adenine; dNTP, deoxyribonucleoside triphosphate; RCA, rolling circle amplification.

Efforts during the past decade revealed three major error sources, with library construction representing the predominant error source, followed by sequencing and DNA damage (**Figure 1**A) [20–22].

### Errors generated during library construction

DNA amplification is widely used in sequencing library construction, and diverse amplification methods introduce errors with variable extents. These methods can be roughly divided into two categories: exponential amplification by polymerase chain reaction (PCR), and linear amplification which mainly includes rolling circle amplification (RCA) and transcription (Figure 1B). PCR potentially generates errors in each amplification cycle, and these errors would be passed on in subsequent cycles. By contrast, linear amplification may also generate errors in each cycle, but these errors will not propagate in the subsequent cycles [15,17,23,24]. Therefore, the errors in the amplification products are independent of each other, making linear amplification a much better option compared to PCR. Notably, in a broader context, strand displacement can be considered a form of linear amplification, albeit limited to a one-copy increase in the copy number [19,25]. Despite the development of linear amplification techniques, PCR is still widely used in two steps of the sequencing process, *i.e.,* library preparation and sequencing cluster amplification (Figure 1A) [1,2]. Errors generated in PCR are caused by limited DNA polymerase fidelity and are further exaggerated by the aforementioned exponential amplification process. For example, the data quality is improved from Q30 (error rate of 10^−3^) to Q40 (error rate of 10^−4^) with the PCR-free library preparation [26].

Notably, during the end repair and A-tailing process (involving the addition of A to connect sequencing adaptors) of library construction, the limited accuracy of the DNA polymerase can induce base errors (Figure 1C). These errors not only produce error-prone 5′ read terminals but also elevate the overall error rate within the DNA library [9,14,25,27–29]. Specifically, single-strand errors can undergo propagation as double-strand errors during routine end repair or A-tailing procedures. For internal errors, strand extension may occur when adjacent nicks are present in the complementary strand during end repair or A-tailing, resulting in error propagation to the complementary strand (Figure 1C). Similarly, terminal errors can also be copied during end repair or A-tailing.

### Sequencing-related errors

The Illumina sequencing-related errors can be roughly divided into eight types [14,30–39]: (1) amplification errors emerging in the generation of sequencing clusters by the aforementioned PCR from a single DNA molecule; (2) errors associated with color or laser cross-talk because of the overlap of the excitation and emission spectra of various fluorophores employed for reading the integrated bases; (3) errors caused by noise arising from cross-talk between neighboring clusters or dephasing (inconsistent pace of DNA synthesis) within the same cluster; (4) errors caused by optical duplication when the same cluster is imaged twice, or DNA amplicons in adjacent clusters are redundantly generated from the same single-strand template; (5) demultiplexing errors arising from misclassification of reads from one sample into another when multiple samples are pooled together for sequencing; (6) errors caused by the unremoved adapter sequences; (7) errors generated by incorrect base-calling; and (8) biased errors caused by special sequence motifs.

Errors in Illumina reads exhibit non-random distributions, with four discernible patterns: (1) the initial bases frequently exhibit errors due to inefficient DNA end repair and the presence of unremoved adapter sequences (Figure 1C and D) [9,14,25,27,28,36,37]; (2) with the increase of base position, later sequenced bases exhibit a heightened error rate, attributed to factors such as DNA strand damage (terminated extension), asynchronous DNA extension, and increased background noise [27,28,31]; (3) in paired-end sequencing, the later sequenced read 2 demonstrates a higher overall error rate than read 1, primarily attributed to the increasing interference between fluorescent signals and escalating background noise [27,28,39]; and (4) DNA libraries with longer insert sizes are associated with a greater error rate (Figure 1D) [27], possibly due to the tendency of longer library fragments to disrupt neighboring sequencing clusters.

### DNA damage

The generation of a DNA sequencing library usually depends on multiple procedures: DNA extraction, fragmentation, end repair, A-tailing, and so on (Figure 1A and C) [22]. Throughout these procedures, DNA damage may be induced by various operations, including heating and oxidation caused by chemical reagents [40–43]. The top two most commonly observed forms of damage include cytosine (C) to thymine (T) substitution, which occurs by C deamination induced by heating; and guanine (G) to T substitution, which occurs by G oxidation (8-oxoG) (Figure 1E) [23]. Consistently, one study showed that error rates with and without heating cycles were 10^−5^ and 10^−7^, respectively [42].

## Twelve strategies for mitigating sequencing errors

Two types of strategies have been applied to control sequencing errors, *i.e.*, error avoidance and error correction (Figure 1F). Each type of strategy consists of six subtypes.

### Error avoidance

With error sources identified, corresponding strategies could be implemented to avoid errors.

For PCR, high-fidelity enzymes can improve sequencing accuracy (A_H_) (Figure 1F). It is reported that the fidelity of Q5 DNA polymerase is higher than other frequently used DNA polymerases, and the adoption of Q5 DNA polymerase reduces errors by one order of magnitude. Additionally, as previously mentioned, reducing PCR cycles or omitting PCR during library construction (A_A_) can also reduce errors [22,44].

To mitigate errors introduced during end repair and A-tailing, blunting DNA with exonuclease during end repair, employing deoxyadenosine triphosphate (dATP) and ddBTP [2′,3′-dideoxycytidine 5′-triphosphate (ddCTP), 2′,3′-dideoxyguanosine 5′-triphosphate (ddGTP), or 2′,3′-dideoxythymidine 5′-triphosphate (ddTTP)] to hinder strand extension in A-tailing, and excising error-prone terminal sequences during mutation calling significantly lower the error rate (A_B_ and A_D_) (Figure 1C and F) [9,14,29].

Regarding DNA damage, errors can be minimized by using restriction enzymes to digest DNA, or avoiding heating and using oxidative reagents (A_M_) [9,29]. Furthermore, uracil-DNA glycosylase (UDG) and formamidopyrimidine DNA glycosylase (Fpg) have been used to remove the aforementioned C-to-T and G-to-T errors, respectively (A_R_) (Figure 1E) [23,25].

### Error correction

Three strategies can correct errors after sequencing, *i.e.*, consensus sequencing, double-strand correction, and validation between paired reads (Figure 1F). As the most popular strategy, consensus sequencing can be further divided into methods that depend on genome mapping position (C_CG_), barcoding (C_CB_), and the aforementioned RCA (C_CR_) or transcription-based linear amplification (C_CT_) (Figure 1F). Among these four strategies, the genome mapping position strategy is most often used, which uses genomic position information as an *in silico* barcode [5,6,9,13–19]. This strategy assumes that reads mapped to the same position are derived from the same DNA molecule. Such an assumption is especially true for libraries with limited DNA input, since the chance of two random DNA fragments sharing the same position is lower. Barcoding is similar to the genome mapping position strategy except that a DNA barcode sequence replaces the mapping position. Barcode or unique molecular identifier (UMI) was initially used for transcriptome sequencing and local assembly of NGS data in 2010 [45,46] and soon adapted for rare mutation calling via consensus sequencing in 2011 [47,48]. This strategy allows each single DNA molecule to be tagged with a unique DNA barcode sequence and thus ensures that the PCR amplification products from the same DNA molecule are grouped by molecular identity. As previously discussed, RCA linearly amplifies the same circular DNA template to generate linked tandem copies; thus, the consensus sequence is free from exponentially accumulated errors caused by PCR [17,23,24]. The transcription-based linear amplification strategy is analogous to RCA except that multiple linear copies with independent error distributions are generated by amplifying the same DNA molecule through transcription and reverse transcription [15]. It is noteworthy that linear amplification mitigates amplification bias to a limited extent, representing a substantial reduction compared with the exponential amplification inherent in PCR. However, complete elimination remains unachievable.

Double-strand correction (C_D_) (Figure 1F) is the second most popular strategy [9,13,14,16,18,19,49]. This approach takes advantage of the complementary property of double-strand DNA: the error at one strand can be corrected by the corresponding reverse strand [49]. For example, if one strand of double-strand DNA has an error caused by DNA damage, library amplification, or sequencing, the correct information from the other strand helps fix this error.

Finally, the validation between paired reads strategy (C_V_) (Figure 1F) is designed for sequencing libraries with short insertion size. With this strategy, the overlapping sequence information provided by paired reads can be used to correct the dephasing-associated errors: the error-resistant 5′ segment of read 1 can be used to correct the errors harbored by the error-prone 3′ segment of read 2, and *vice versa* (Figure 1D) [14,25]. Notably, in contrast to double-strand correction, this strategy relies solely on the information from a single strand.

## Eleven representative high-fidelity NGS whole-genome sequencing methods

Since 2011 [47], dozens of sequencing methods have been developed [5,6,9,13–19], which took advantage of the two types of strategies (error avoidance or error correction) to lower the error rates (Figure 1F). We reviewed 11 representative methods implementing distinct combinations of strategies (**Table 1**). It should be noted that each method consists of complicated experimental or computational procedures and even the implementation of the same strategy may be somewhat variable across methods. For additional details, we refer readers to individual publications (Table 1) or the two previous reviews [5,6]. To zoom into fundamental differences across methods, we organize these methods into three groups according to library amplification modes because errors are mainly generated in amplification. These groups include four methods with PCR, six hybrid methods with both linear amplification and PCR, and one amplification-free method (Table 1). We will introduce these individual methods by summarizing how the 12 strategies in Figure 1F are combined to achieve high accuracy. Because the genome mapping position strategy is applied in every method, we will not describe it in the following analysis unless necessary.

**Table 1:** Eleven representative high-fidelity NGS WGS methods

Group	Method	Strategy	Error rate	Linked copies	Insertion size	DNA input
Method with PCR	DupSeq [49]	C_CB_+C_CG_+C_D_	10^−6*^	No	Normal	Low
	BotSeqS [18]	C_CG_+C_D_	10^−7^	No	Normal	Low
	META-CS [16]	C_CB_+C_CG_+C_D_+A_H_+A_D_	10^−8^	No	Normal	Single-cell
	NanoSeq [9]	C_CB_+C_CG_+C_D_+A_H_+A_M_+A_B_	10^−9^	No	Normal	Low
Hybrid method with both linear amplification and PCR	CircSeq [23]	C_CR_+C_CG_+A_R_	10^−6^	Yes	Short	Low
	CypherSeq [24]	C_CR_+C_CG_+C_D_	10^−6*^	No	Normal	Low
	SMM-seq [17]	C_CR_+C_CG_+C_D_+C_CB_+A_D_	10^−7*^	No	Normal	Low
	o2n-seq [25]	C_V_+C_CG_+A_R_+A_D_	10^−8^	Yes	Short	Low
	CODEC [19]	C_CB_+C_CG_+C_D_+A_D_+A_B_	10^−8*^	Yes	Short	Low
	LIANTI [15]	C_CT_+C_CG_+A_R_	10^−6^	No	Normal	Single-cell
Amplification-free library preparation method	PECC-Seq [14]	C_V_+C_CG_+C_D_+A_D_+A_A_	10^−7^	No	Short	High

*Note*: The “Strategy” column shows the corresponding strategy combination for each method with the abbreviation of different strategies same as in Figure 1F: A_H_, high-fidelity DNA polymerases; A_A_, amplification-free library preparation; A_B_, blocking errors from end repair and A-tailing; A_D_, deletion of error-prone terminal sequences; A_M_, minimization of DNA damages; A_R_, removal of DNA damages; C_CG_, genome mapping position; C_CB_, barcoding; C_CR_ or RCA, rolling circle amplification; C_CT_, transcription-based linear amplification; C_D_, double-strand correction; C_V_, validation between paired reads. Within each group, the methods are sorted by publication time and by the relationship across methods. Numbers marked with “*” represent the sensitivity or the detection limit of the corresponding methods while the actual error rate was not reported in the original paper. The “Linked copies” column means that the amplified copies are physically linked within one amplicon. The short insertion size indicates an insertion size of about 100–170 bp, which is shorter than that of the other methods or the conventional Illumina sequencing. The last column shows the DNA input demand of different methods. WGS, whole-genome sequencing; NGS, next-generation sequencing; PCR, polymerase chain reaction; DupSeq, duplex sequencing; BotSeqS, bottleneck sequencing system; META-CS, multiplexed end-tagging amplification of complementary strands; NanoSeq, nanorate sequencing; CircSeq, circle sequencing; SMM-seq, single-molecule mutation sequencing; CODEC, concatenating original duplex for error correction; LIANTI, linear amplification via transposon insertion; PECC-Seq, paired-end and complementary consensus sequencing.

### Methods with PCR

Despite the errors induced by exponential amplification (Figure 1B), PCR is still widely used even in high-fidelity sequencing methods. For each of the following four methods, dedicated procedures are needed to avoid or correct errors produced by PCR and other sources. As the pioneering method of the field, duplex sequencing (DupSeq) [49] combines barcoding and double-strand correction strategies (Table 1), which can independently reduce errors. Because DupSeq can detect the mutation rate of a plasmid (10^−6^) [5,49], its actual error rate should be lower than this sensitivity. Widely adopted in academic and commercial domains, DupSeq fosters subsequent methodological advancements [5,6]. Specifically, the bottleneck sequencing system (BotSeqS) [18] is similar to DupSeq but without the barcoding strategy (Table 1). It approaches an error rate of 10^−7^ [9]. Similarly, both the multiplexed end-tagging amplification of complementary strands (META-CS) [16] and nanorate sequencing (NanoSeq) [9] are built upon DupSeq. They also adopt high-fidelity polymerases to reduce amplification errors. META-CS achieves an error rate of 10^−8^ by employing the extra strategy of deleting error-prone terminal sequences. In contrast, NanoSeq achieves the lowest reported error rate in NGS at 10^−9^, attributed to the integration of two additional strategies: minimizing DNA damages and blocking errors from end repair and A-tailing (Table 1).

### Hybrid methods with both linear amplification and PCR

To reduce errors, PCR could be replaced with linear amplification (Figure 1B). Six methods utilize linear amplification for DNA amplification, followed by PCR to generate the final sequencing library.

Circle sequencing (CircSeq) is the first method to apply the RCA strategy for consensus sequencing (Figure 1B) [23]. By generating a consensus based on tandemly linked copies from the same DNA molecule and removing DNA damage, CircSeq [23] reduces the error rate to 10^−6^ (Table 1). Two related methods were developed later. CypherSeq replaces the strategy of removing DNA damage with the double-strand correction strategy facilitated by plasmid-based double-strand DNA circularization. It has successfully detected a yeast mutation rate of 10^−6^ [24]. Single-molecule mutation sequencing (SMM-seq) [17] uses a hairpin structure adapter with a unique barcode to prepare the circular DNA sequencing library. By combining RCA, double-strand correction, barcoding, and deletion of error-prone terminal sequences, the mutation rate of the IMR90 cell line is quantified as 10^−7^ by SMM-seq (Table 1). Notably, similar to DupSeq, the actual error rate of the latter two methods is unknown but is expected to be lower than the reported mutation rates.

In contrast to CircSeq, CypherSeq, and SMM-seq, o2n-seq [25] and Concatenating Original Duplex for Error Correction (CODEC) [19] implement strand displacement to generate linked copies. Specifically, with strand displacement, o2n-seq obtains two tandem copies from one circular single-strand DNA molecule. This creative strategy has been only applied in o2n-seq, possibly because of the intrinsic complexity of the experimental procedure. By further integrating additional strategies, including validation between paired reads, removal of DNA damages, and deletion of error-prone terminal sequences (Table 1), o2n-seq reaches an error rate of 10^−8^. It is the most precise method that does not rely on the two most popular strategies, *i.e.,* barcoding and double-strand correction. CODEC employs a dual-barcoded long adapter ligated to both ends of the same double-strand DNA. Subsequent strand displacement results in two copies of the original DNA within each strand, containing both forward and reverse strand sequence information. Incorporating additional strategies such as barcoding, double-strand correction, deletion of error-prone terminal sequences, and blocking errors from end repair and A-tailing (Table 1), CODEC determines a human germline mutation rate at the 10^−8^ level [19].

Different from these five methods adopting RCA or strand displacement techniques, Linear Amplification via Transposon Insertion (LIANTI) [15] implements a transcription-based linear amplification strategy (Figure 1B). With the Tn5 transposition reaction, genomic fragments are efficiently tagged with the T7 promoter sequence and linearly amplified via transcription and reverse transcription (Table 1). LIANTI achieves an error rate of 10^−6^ [15]. Notably, apart from LIANTI, several single-cell genome sequencing methods based on multiple displacement amplification (MDA) have emerged, including emulsion MDA (eMDA) [50], digital droplet MDA (ddMDA) [51], TruePrime [52], and the primary template-directed amplification (PTA) methods [53]. In contrast to LIANTI, which achieves multiple rounds of transcription to amplify the same DNA template, MDA-related approaches amplify DNA using random short primers, resulting in amplicons that generally lack the same chromosomal coordinates and cannot be traced back through genome mapping position or barcoding strategies. In other words, while it is feasible to generate a consensus sequence based on genome mapping position in LIANTI, such an approach does not apply to MDA-related methods.

Following initial linear amplification of DNA, all these six methods use PCR to generate the final sequencing libraries.

### An amplification-free library preparation method

Paired-End and Complementary Consensus Sequencing (PECC-Seq) [14] combines multiple strategies, including validation between paired reads (Figure 1F), double-strand correction, deletion of error-prone terminal sequences, and amplification-free library preparation (Table 1). Its error rate is 10^−7^. In addition to amplification-free operation, a distinguishing feature of PECC-Seq is its utilization of both validation between paired reads and double-strand correction strategies, whereas all other methods employ just one of these strategies (Table 1).

## Three trends emerging in developing high-fidelity NGS methods

In reviewing high-fidelity sequencing methods, three discernible trends emerge: the gradual improvement of accuracy through the implementation of an increasing number of strategies, a dual motivation including cost reduction and accuracy enhancement, and divergence of methods based on varied application contexts.

### An increasing number of strategies have been integrated into newer methods

Possibly because distinct strategies are complementary to each other, newer methods tend to integrate more strategies to gain higher accuracy (Table 1). NanoSeq employs six strategies, achieving the lowest reported error rate at 10^−9^ level [9]. CODEC, employing up to five strategies, likely attains comparable accuracy, as it quantifies a mutation rate of 10^−8^ level [19]. Notably, error reduction mainly relies on error correction strategies, whereas error avoidance strategies help to further increase accuracy. Methods with only error correction strategies already show error rates of around 10^−7^–10^−6^, and the incorporation of avoidance strategies further lowers the error rate to 10^−9^–10^−7^ (Table 1).

### Method developments have been driven by cost reduction rather than solely focusing on accuracy improvement

While newer methods generally exhibit higher accuracy (Table 1), ongoing methodological advancements are not solely motivated by enhancing fidelity. Cost remains a crucial consideration in driving these developments.

The core of high-fidelity sequencing is to generate precise reads with redundant sequencing, which however leads to an increase in cost. Specifically, although all 12 strategies are used at least one time in one method (Figure 1F; Table 1), consensus sequencing-derived strategies (especially genome mapping position, barcoding_,_ and RCA) and double-strand correction are relatively more often used. Double-strand correction also represents a consensus sequencing strategy in which the consensus is inferred based on double strands of DNA. The aforementioned consensus sequencing-derived strategies do not have this explicit requirement, and the corresponding consensus may only use the information of one strand. Nonetheless, both consensus sequencing and double-strand correction represent redundant sequencing, in which the accuracy is increased at the cost of higher sequencing depth of the same DNA molecule [6].

**Figure 2:**
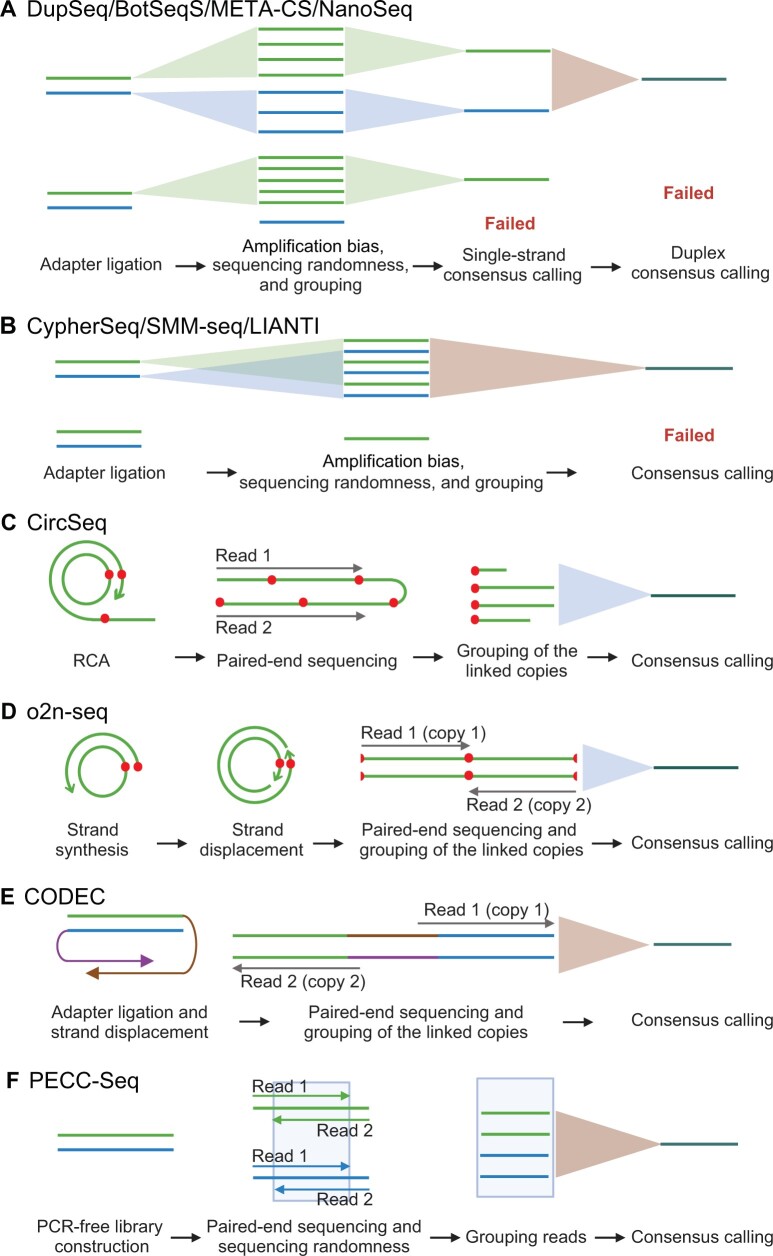
Schematic diagram of consensus calling across different methods **A**. DupSeq, BotSeqS, META-CS, and NanoSeq. These methods adopt two rounds of duplex consensus calling, for both of which amplification bias and sequencing randomness may lead to failure of consensus sequence generation. **B**. CypherSeq, SMM-seq, and LIANTI. Only one round of consensus calling was performed. **C**. CircSeq. **D**. o2n-seq. **E**. CODEC. CircSeq, o2n-seq, and CODEC rely on the linked copies to improve data efficiency. As shown in Figure 1B, the red dots show the boundary of each copy. CircSeq generates multiple copies for one DNA molecule, while o2n-seq and CODEC only generate two copies. Notably, CircSeq used 250 bp paired-end sequencing in which one read could be long enough to cover one fragment more than one time. **F**. PECC-Seq. With amplification-free library preparation and consensus calling by overlapping reads, PECC-Seq reaches a middle data efficiency. For some methods (*e.g*., DupSeq), read grouping is achieved through barcodes and/or mapping positions, while for the other methods, grouping is guided by only mapping positions (see also Table 1). In (D) and (F), validation or overlapping between paired reads is also demonstrated. For the consensus calling process, the light blue triangle represents the consensus sequence generated from single-strand template DNA, while the light brown triangle represents the consensus sequence generated from double-strand template DNA. DupSeq, duplex sequencing; BotSeqS, bottleneck sequencing system; META-CS, multiplexed end-tagging amplification of complementary strands; NanoSeq, nanorate sequencing; CircSeq, circle sequencing; SMM-seq, single-molecule mutation sequencing; CODEC, Concatenating Original Duplex for Error Correction; LIANTI, Linear Amplification via Transposon Insertion; PECC-Seq, Paired-End and Complementary Consensus Sequencing.

The key parameter that determines the cost is data efficiency, which is defined as the proportion of final consensus bases to all sequencing bases [23,25]. If a method could generate a consensus read for most (if not all) sampled DNA molecules with relatively fewer raw reads, it would get a higher data efficiency. However, in practical applications, data efficiency faces constraints attributed to three factors (**Figure 2**; **Table 2**): (1) the consensus generation process; (2) amplification bias introduced by techniques like PCR, resulting in disparate copy numbers for different DNA molecules; and (3) sequencing randomness, leading to over-representation of certain amplified or unamplified DNA molecules along with under-representation or non-representation of others during sequencing.

**Table 2:** Data efficiency of different NGS methods and major factors affecting data efficiency

Method	Data efficiency	Factor affecting data efficiency
DupSeq	Low	Two rounds of consensus calling;
BotSeqS		strong amplification bias of PCR;
META-CS		sequencing randomness
NanoSeq		
PECC-Seq	Middle	One round of consensus calling;
		amplification free;
		sequencing randomness
CypherSeq	Middle	One round of consensus calling;
SMM-seq		moderate amplification bias;
LIANTI		sequencing randomness
CircSeq	High	One round of consensus calling;
o2n-seq*		moderate amplification bias;
CODEC*		linked copies

*Note*: * Both o2n-seq and CODEC methods can generate the consensus sequence with only one pair of reads in one round of consensus calling.

Specifically, the pioneering high-fidelity sequencing method, *i.e.*, DupSeq, applies two rounds of consensus calling where primary consensus sequences from both the forward and reverse strands were combined to generate the final consensus (Figure 2A; Table 2). If one strand is not amplified or not sampled multiple times and no primary consensus sequence is generated, data from both strands would be wasted even if the primary consensus sequence is generated for the other strand. The two rounds of consensus calling operation have been followed by BotSeqS, META-CS, and NanoSeq (Figure 2A). Together with amplification bias and sequencing randomness, these four methods are expected to have low data efficiency (Table 2).

In contrast, the remaining seven methods employ a single round of consensus calling and substitute PCR with linear amplification or adopt an amplification-free library construction approach (Figure 2B–F; Table 1), resulting in enhanced data efficiency. Notably, by mitigating sequencing randomness and/or amplification bias, CircSeq, o2n-seq, and CODEC stand out for their significantly higher data efficiency compared with PECC-Seq, CypherSeq, SMM-seq, and LIANTI (Figure 2; Table 2) [6].

To address sequencing randomness, two strategies have been implemented: (1) physically linking amplified copies within one amplicon for CircSeq, o2n-seq, and CODEC (Figure 2C–E), with linked copies sequenced simultaneously through paired reads, ensuring redundancy for each molecule; and (2) employing overlapping reads for validation between paired reads, as seen in o2n-seq and PECC-Seq (Figure 2D and F). Similarly, two strategies control amplification bias: (1) PECC-Seq, preventing amplification for generating redundant copies [14], utilizes two read pairs to separately cover both strands of any single DNA molecule (Figure 2F); and (2) o2n-seq and CODEC restrict the amplified copy number precisely to two. In this regard, it is conceivable that o2n-seq and CODEC exhibit relatively high data efficiency compared to CircSeq [25], despite all three methods implementing a single round of consensus calling and generating linked reads (Table 2).

Notably, to achieve multiple copies in one amplicon or generate two reads in one overlapping pair, the library sizes of CircSeq, o2n-seq, CODEC, and PECC-Seq (100–170 bp) are notably shorter than those of other methods (Table 1).

### The application context is diverging across methods

The differences in accuracy, cost, and underlying design details make these methods diverge in their application context. Highly precise NanoSeq and CODEC would be considered for studies focusing on ultra-rare variants, while methods with high data efficiency such as CircSeq, o2n-seq, and CODEC would be preferred if the budget is tight. The specific design also matters in at least four aspects (Table 1). First, META-CS and LIANTI are dedicated to single-cell sequencing. Second, PECC-Seq is suitable for studies with a large DNA input. Third, if a study is interested in mutations in relatively repetitive regions, all these NGS-based methods would be confounded by multi-mapping issues due to short read length. Among them, CircSeq, o2n-seq, CODEC, and PECC-Seq are particularly less suitable given their small library size (Table 1). Finally, for projects focusing on mutational signatures (*e.g.*, the non-random distribution of nucleotide substitutions), relatively more precise methods (NanoSeq, CODEC, or o2n-seq) (Table 1) would be preferred.

## Eight representative high-fidelity long-read whole-genome sequencing methods

As mentioned above, short reads make NGS incapable of resolving repetitive regions. Thus, long-read sequencing technologies represented by Pacific Biosciences (PacBio) Single Molecule Real-Time (SMRT) and Oxford Nanopore Technologies (ONT) are blooming in recent years in fields such as *de novo* genome assembly, structural variation analyses, and full-length isoform analyses [54–58]. Although the sequencing reads of these two platforms showed a high error rate (10% or even higher) in the past, they are now much more precise (0.1% or even lower). In addition to the improvement of base calling algorithms [55,59,60], experimental strategies especially amplification-free library preparation, RCA, or double-strand correction have contributed to the observed increase in fidelity [29,59,61–63].

**Figure 3:**
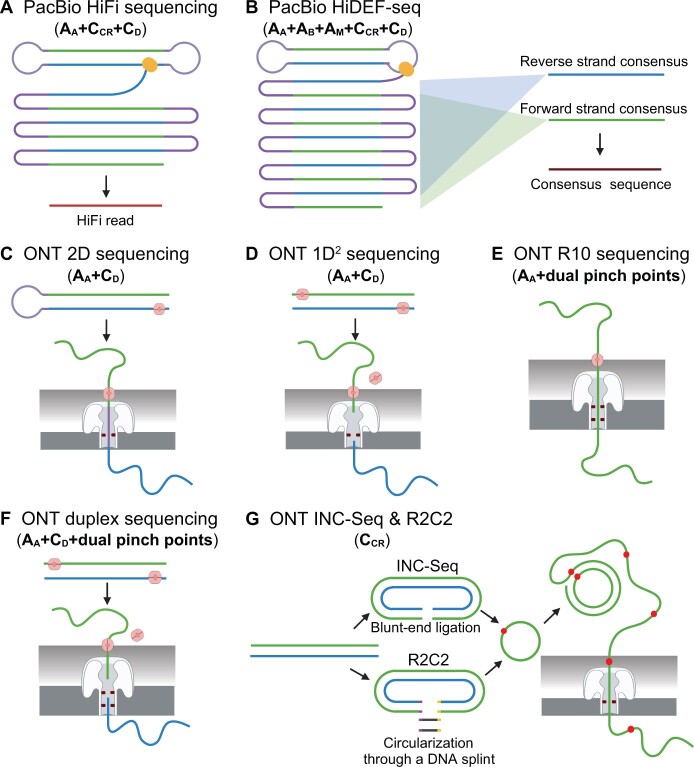
Schematic diagram of high-fidelity long-read sequencing methods **A**. PacBio HiFi sequencing. HiFi sequencing applies amplification-free library preparation, RCA consensus sequencing, and double-strand correction strategies to generate the final HiFi read (in red). **B**. PacBio HiDEF-seq. HiDEF-seq implements two additional strategies including error blockage from end repair and A-tailing and minimization of DNA damages. It also applies a DupSeq-like single-strand and double-strand consensus calling framework (Figure 2A). In (A) and (B), a single DNA molecule is circularized via two hairpin adaptors where the two strands are marked in green and blue, respectively. These strands would be presented as linked multicopy sequences in one amplicon. The yellow dot marks DNA polymerase. **C**. ONT 2D sequencing. **D**. ONT 1D^2^ sequencing. **E**. ONT R10 sequencing. **F**. ONT duplex sequencing. The motor protein is depicted as a filled pink circle, whereas the sequencing readers are represented as pairs of dark red rectangles. Notably, both ONT R10 and duplex sequencing employ two readers (dual pinch points). **G**. ONT INC-Seq (upper) and R2C2 (lower). Both methods use RCA to amplify a circular DNA to obtain multiple linked copies within one amplicon. With a dedicated DNA splint for ligation, R2C2 has a higher DNA circularization efficiency than INC-Seq. The red dot marks the boundary between different copies. PacBio, Pacific Biosciences; HiFi, high-fidelity; HiDEF-seq, Hairpin Duplex Enhanced Fidelity Sequencing; ONT, Oxford Nanopore Technologies; INC-Seq, Intramolecular-ligated Nanopore Consensus Sequencing; R2C2, Rolling Circle Amplification to Concatemeric Consensus.

Specifically, the PacBio platform has two running modes including continuous long read (CLR) mode and high-fidelity (HiFi) mode with the latter being much more precise [64]. In the case of CLR, the extended template length leads to a reduced number of copies or passes of the same DNA template, resulting in an error rate ranging from 0.08 to 0.13 [64]. Complementing the amplification-free library preparation in CLR, HiFi sequencing incorporates RCA and double-strand correction to further diminish the error rate (Figure 1E and **Figure 3**A) [29,61,65]. Together with an increasing number of passes, the error rate of HiFi reaches a plateau between 0.1% and 0.01% [60,65]. In contrast to conventional HiFi sequencing, the recently reported Hairpin Duplex Enhanced Fidelity Sequencing (HiDEF-seq) employs restriction enzymes for genomic DNA fragmentation [29]. It selectively targets shorter DNA fragments to ensure an independent sequencing of both forward and reverse strands, each with a coverage of at least five-fold. HiDEF-seq implements a consensus calling strategy akin to DupSeq, resulting in the generation of single-strand and double-strand consensus sequences (Figures 2A and 3B). Utilizing amplification-free library preparation, blocking errors from end repair and A-tailing, minimizing DNA damages, implementing RCA, and incorporating double-strand correction (Figure 1F), HiDEF-seq has achieved the lowest reported double-strand error rate, ranging from 10^−17^ to 10^−16^ [29]. While achieving this unprecedented accuracy, HiDEF-seq has significantly decreased total data production by 5-fold to 10-fold compared with regular HiFi sequencing, attributable to the higher level of data redundancy facilitated by an increased number of passes.

Compared to the PacBio platform, the inherently higher raw error rate of the ONT platform has driven the development of relatively more high-fidelity methods, as depicted in Figure 3C–G [54,55,59,62,63,66–68]. In addition to the previously mentioned amplification-free library preparation, double-strand correction, and RCA, a unique strategy named dual pinch point is designed for ONT, enabling the sequencing of a single DNA molecule twice. Specifically, ONT 2D, 1D^2^, and duplex sequencing incorporate amplification-free library preparation and double-strand correction strategies. In ONT 2D sequencing, the two strands are linked via a hairpin adapter, while they remain separate in ONT 1D^2^ and duplex sequencing (Figure 3C, D, and F). Moreover, both ONT Intramolecular-ligated Nanopore Consensus Sequencing (INC-Seq) and Rolling Circle Amplification to Concatemeric Consensus (R2C2) include RCA in the library preparation, with the latter optimizing DNA circularization efficiency through the DNA splint-mediated circularization method (Figure 3G). The dual pinch point strategy, a recent innovation facilitated by ONT R10 flow cells, elevates median accuracy to 99% (Figure 3E). Importantly, ONT duplex sequencing also leverages these latest R10 flow cells, coupled with updated base callers, resulting in ONT reads with 99.9% accuracy (Figure 3F).

Notably, the consensus read of most long-read sequencing methods (PacBio HiFi or ONT) only reaches an error rate of 0.1% for single-molecule DNA sequencing, which is several magnitudes higher than that of NGS (Table 1). Such a dramatic difference is rooted in the intrinsic difference in read length and data throughput between long-read and short-read sequencing. Fragments with a dozen kilobases or even longer lengths are preferred in long-read sequencing. These fragments are more difficult to amplify, which makes amplification-based redundant sequencing less feasible. An even more constraining factor is the relatively lower throughput of long-read sequencing compared with NGS. Taking PacBio as an example, its widely-used Sequel IIe cell only consists of 8 million SMRT wells and usually generates 4 million HiFi reads (> 300 dollars per million reads) [64]. The recently introduced PacBio Revio sequencing, featuring 25 million SMRT wells, has successfully lowered the cost to approximately 100 dollars per million reads; however, this remains relatively high [69]. To detect the rare mutations (frequency < 0.1%), such a low throughput is far from enough. More reads are demanded, leading to a formidable cost. By contrast, high-fidelity NGS usually generates hundreds of millions of raw reads, enabling downstream calling of enough consensus sequences. Such a high amount could be only achieved with high throughput and low cost of NGS (0.1–1 dollar per million reads) [70].

In summary, aside from HiDEF-seq, which demonstrates an exceptional accuracy in the range of 10^−17^ to 10^−16^ [29], single-molecule long-read sequencing methods reach a consensus accuracy of 99.9%. This level of accuracy is only on par with the raw reads obtained from NGS [64]. Therefore, long reads are now routinely used for genome assembly and detection of structural variants and high-frequency point mutations [54,71], rather than the detection of low-frequency (< 0.1%) point mutations.

## Two perspectives

Given the trends of methodological development in the past decade, we anticipate that both high-fidelity NGS and long-read sequencing (especially ONT sequencing) could be further enhanced.

### Improving high-fidelity NGS

With the broad usage of consensus sequencing and double-strand correction, their complementary nature, and the limitation of preexisting methods, we expect that future cutting-edge high-fidelity NGS methods may emerge in the following direction. Specifically, although NanoSeq [9] already reaches a high accuracy, it only adopts 6 out of the 12 strategies shown in Figure 1F, and the addition of other strategies may help improve sequencing accuracy or data efficiency. Notably, three strategies (barcoding, genome mapping position_,_ and double-strand correction) are shared between NanoSeq and SMM-seq (Table 1), but SMM-seq also uses RCA. Thus, it is probable that RCA could be integrated into NanoSeq to alleviate amplification bias or sequencing randomness, thereby increasing data efficiency.

### Improving high-fidelity ONT sequencing

As mentioned above, high-fidelity long-read sequencing is conventionally constrained by large fragment size and low data yield. However, we expect significant enhancements owing to the rapidly increasing throughput. While the PacBio platform with HiDEF-seq demonstrates an impressively low error rate (10^−17^–10^−16^) [29], the state-of-the-art ONT duplex sequencing only reaches 0.001, indicating room for substantial improvement in base accuracy. Present ONT sequencing methods involve amplification-free library preparation, RCA, or double-strand correction (Figure 3C–G). Combining RCA and double-strand correction strategies or incorporating additional approaches outlined in Figure 1F, such as UMI or barcoding (Figure 1F), holds promise to further improve accuracy. Interestingly, barcoding has already been implemented in targeted ONT sequencing, achieving an error rate of 0.004 [72]. Presumably, akin to PacBio or NGS high-fidelity sequencing (Figure 3A and B; Table 1), a combination of these strategies will yield more precise ONT reads (error rate < 0.001).

## Discussion

In this review, we summarized three primary sequencing errors, presented 12 error reduction strategies, and analyzed 11 high-fidelity short-read sequencing methods alongside eight long-read sequencing methods. Additionally, we provided two perspectives on future developments. These efforts offer a systematic guide for those interested in understanding error sources, addressing them, utilizing high-fidelity sequencing techniques, and innovating new methods.

Specifically, we first generated a simplified error classification and error reduction framework, facilitating an accessible overview. Sequencing errors can manifest at various stages within the whole process (Figure 1A). To streamline the inherent physiochemical intricacies contributing to errors, we categorized them as DNA damage, amplification errors, or sequencing mistakes. Similarly, we condensed the 12 complicated error reduction strategies into two groups: error avoidance and error correction (Figure 1F). The former mitigates errors arising from damages or amplification, while the latter leverages redundant sequencing to generate consensus reads of higher quality compared with raw reads. Importantly, these two categories of strategies may exhibit some overlap, as exemplified by techniques like RCA or transcription-based amplification, which not only provide a consensus sequencing method but also reduce errors compared with widely used exponential amplification (PCR) (Figure 1B).

Second, we provided a comprehensive and practical guideline for users of high-fidelity sequencing. While we could not exhaustively cover all relevant methods, we discussed the majority of published approaches. By anchoring all methods according to their major error reduction strategies, we elucidated their mechanisms for achieving high fidelity (Figures 1F and 3; Table 1). Furthermore, we identified key parameters, including data efficiency and application context (Figure 2; Tables 1 and 2), allowing readers to evaluate the advantages and disadvantages of different methods for specific applications.

Finally, we highlighted promising directions for enhancing both high-fidelity short-read and long-read sequencing. The complementarity of diverse strategies presents opportunities for further optimization in generating precise reads with heightened data efficiency or reduced cost. Notably, while fidelity and cost are crucial parameters, the field may evolve in other dimensions. For instance, with the recent introduction of single-cell long-read sequencing methods [73–75], current high-fidelity long-read sequencing techniques such as PacBio HiDEF-seq or ONT duplex sequencing could be tailored for single-cell applications.

In conclusion, ongoing methodological developments yield precise short-read and long-read DNA sequencing data. This progress has and will continue to enhance the generation of more accurate genetic profiles in heterogeneous organismal or environmental samples, further advancing fields such as somatic or germline mosaicism, cancer genomics, antibiotic resistance, and forensics [5–8].

## CRediT author statement

**Hangxing Jia:** Conceptualization, Investigation, Visualization, Writing – original draft, Writing – review & editing, Project administration. **Shengjun Tan:** Visualization, Writing – review & editing. **Yong E. Zhang:** Conceptualization, Supervision, Writing – review & editing, Project administration, Funding acquisition. All authors have read and approved the final manuscript.

## Competing interests

The authors have declared no competing interests.
